# Public health supply chain for iron and folic acid supplementation in India: Status, bottlenecks and an agenda for corrective action under Anemia Mukt Bharat strategy

**DOI:** 10.1371/journal.pone.0279827

**Published:** 2023-02-24

**Authors:** Khobaib Ahmad, Jitendra Singh, Ruby Alambusha Singh, Abhimanyu Saxena, Mini Varghese, Sebanti Ghosh, Sumitro Roy, Kapil Yadav, William Joe, Narendra Patel

**Affiliations:** 1 Institute of Economic Growth, Delhi University Enclave, North Campus, Delhi, India; 2 Department of Operational Research, University of Delhi, Delhi, India; 3 Health System Strengthening, United Nations Development Programme (UNDP), Delhi, India; 4 Nutrition, Nutrition International (NI), Delhi, India; 5 Alive & Thrive Initiative, New Delhi, India; 6 Nutrition, IPE Global Limited, Ranchi, Jharkhand, India; 7 Centre for Community Medicine (CCM), All India Institute of Medical Sciences (AIIMS), Delhi, India; VART Consulting PVT LTD, INDIA

## Abstract

**Purpose:**

The IFA supplementation program under the Anemia Mukt Bharat (AMB) program is one of the most ambitious nutrient supplementation programs in India. The delivery of services often suffers due to frequent stock outs and shortages. It is critical to understand the bottleneck in the supply chain adversely affecting the performance and coverage of the program. The paper attempts to identify the bottlenecks of the IFA supply chain in key areas of supply chain i.e., forecasting, procurement, warehousing and inventory management, transportation, distribution, logistic information system and suggests a plan of action aimed at ensuring uninterrupted supplies to the end beneficiaries.

**Design/methodology/approach:**

The data source for the present paper is the nationwide IFA Supply Chain Assessment (2018–19) conducted across 29 Indian states with a total of 58 districts, 116 blocks, 232 Sub-Centres, 232 Anganwadi centres and 232 schools covered under the assessment as a multi-partner collaborative initiative. Field insights from supply chain strengthening interventions under different public health programs in India and other developing countries were taken to arrive at corrective actions and recommendations. Findings were disseminated to government and an action plan was suggested for connecting service delivery points through an app-based system, developing a micro plan for ensuring fixed distribution schedule, followed by continuous monitoring and review meetings identified for follow up.

**Findings:**

The average lead time across states was 35 weeks with top three performing states being Goa, Sikkim, and Telangana. The average per unit cost of procurement was Rs 0.35 for IFA Red, Rs 0.25 for IFA Blue, Rs 0.31 for IFA Pink and Rs 7.30 for IFA syrup. Out of the 704 districts in India, only 213 has IFA Red, only 140 had IFA Blue, 152 had IFA Pink and 163 had IFA Syrup available in four quarters of 2018–19. The key issues identified in the assessment were–a lack of standardized forecasting process, absence of inventory management techniques, no fixed distribution schedule, inadequate availability of transport vehicles and an absence of an integrated MIS.

**Originality/value:**

The identification of bottlenecks in the IFA supply chain and its impact on the performance of the supply chain would provide policy guidelines for the government as well as development partner agencies to design an effective and efficient supply chain. It would also enable the policy planners to understand the challenges associated with managing different components of a supply chain, their interrelation and impact on the overall performance of the supply chain. The suggested recommendations would equip program managers with the tool to devise and implement field level solutions.

## Introduction

Anemia is a severe public health problem in India. For decades, the prevalence has remained persistently high across all age-sex groups including women, children, and adolescents [1]. Iron deficiency and insufficient intake of micronutrients such as folate and vitamin B12 are identified as the leading cause of anemia both in India and globally [2–4]. Accordingly, public health efforts have relied extensively on large-scale prophylactic iron and folic acid (IFA) supplementation programs to control anemia. India had launched the National Nutritional Anemia Prophylaxis Program (NNAPP) as early as 1972. However, the program could not make much impact because of multifaceted problems such as poor coverage, substandard drug quality, non-adherence with consumption, poor implementation of the program, inadequate supplies, inadequate funds for overall coverage, lack of training of health functionaries, lack of community participation, lack of program monitoring and supervision [5, 6]. Poor training exposure and organizational support for capacity building attribute to weak knowledge and competency in supply chain functions and undermine the capacity to perform essential supply chain functions [7].

Nevertheless, the Government of India remains committed toward programmatic efforts to address anemia with time-to-time re-strategizing the approach toward anemia prevention and control. This includes landmark initiatives such as the National Nutritional Anemia Control Programme (NNACP) in 1991, National Iron Plus Initiative (NIPI) of 2013 and Intensified National Iron Plus Initiative (INIPI) of 2018. The INIPI is also referred to as Anemia Mukt Bharat (AMB) strategy and is an integral part of the Prime Minister’s Overarching Scheme for Holistic Nutrition in India (POSHAN) *Abhiyaan* [8]. The AMB framework is guided by a 6x6x6 strategy that signifies its focus on six beneficiary groups, six treatment alternatives and six institutional mechanisms. Prophylactic IFA supplementation is an integral part of the 6x6x6 strategy to cover the six beneficiary groups, viz. children (6–59 months), adolescent girls (15–19 years), adolescent boys (10–19 years), women of reproductive age (WRA), pregnant women and lactating mothers. The AMB programme for IFA supplementation serves 26 million pregnant women, 13 million lactating mothers, and 73 million school-age children and adolescents (5–19 years) and 17 million children under of 5–69 months [9].

The delivery of IFA supplementation services under AMB critically depends on the supply chain and the strategy must overcome supply-related challenges and shortcomings to promote effective coverage. The supply chain is unable to provide drugs efficiently and effectively [5, 6]. The delayed procurement and lack of trained human resources are affecting the coverage of the IFA supplies and thus overall supply chain management [10]. Furthermore, generally it suffers from considerable gaps in the distribution process, shortage of drugs at health facilities and lack of availability of IFA for the beneficiaries [11–13]. The supply chain management practices also vary across contexts and geographies [14, 15]. Recognizing this as a serious concern, the Ministry of Health and Family Welfare (MoHFW) has identified strengthening of supply chain and logistics a key institutional mechanism under AMB [16]. Notably, the IFA supply chain is also a marker of performance of the public health supply chain that requires coordination and convergence across line departments such as health, education and women and child development. The AMB strategy, therefore, deserves detailed attention to enable the beneficiaries to receive IFA supplements in time and prevent adverse implications of iron deficiency [17].

Evidence on the performance of the public health supply chain in India is limited, with a few state-level studies as exceptions. A recent cross-sectional, observational, mixed-methods study conducted in Bihar highlighted bottlenecks impacting IFA forecasting, procurement, storage, disposal, lack of personnel, and few training opportunities for key players in the supply chain [11]. A similar study was conducted in Odisha to assess the logistic performance of multiple drug supply chains [18]. A few studies attempt to understand the factors influencing the public health supply chain while some suggest different models for measuring public health supply chain performance [19–24]. The medicines distribution model in Tamil Nadu in India is an example of a high-performance public-sector supply chain achieved through capacity building and management [25]. Some studies also examine the last mile challenges that deprive the beneficiaries of IFA supplements [12, 13].

However, to the best of our knowledge, there is no comparative pan-India evidence to understand the prevalent practices in the IFA supply chain across the different states in India and to identify the bottlenecks associated with the supply chain of each of the states. Against this backdrop, this paper presents the key findings from the nationwide supply chain diagnostic assessment with the following specific objectives: a) to identify the key practices adopted by states in the IFA supply chain; b) to identify bottlenecks in each of the major supply chain functions of forecasting, procurement, warehousing and inventory management, transportation and distribution and logistics management information system, and; c) to develop specific policy recommendations for strengthening of the IFA supply chain. The findings of the paper are expected to improve understanding of the IFA supplementation supply chain across Indian states and can contribute by highlighting the bottlenecks and areas for correction action.

## Data and methods

The nationwide IFA supply chain assessment (2019–20) was conducted across all 29 Indian states. The assessment was supported by the Ministry of Health and Family Welfare, Government of India, and the line departments from the state governments responsible for implementing the AMB strategy. The assessment was led by the Institute of Economic Growth (IEG), Delhi and was conducted in collaboration with six development partners viz. Evidence Action, IPE Global, Nutrition International, UNDP and UNICEF. The study and study protocols were approved by the Ministry of Health and Family Welfare (MoHFW), Government of India. All participants included in the study are adults and were asked for written informed consent for interviews.

For the field-based assessment, two districts from each of the 29 states were selected. *Aspirational* districts were given priority for selection as these districts are specifically focused by the union and the state governments for a quick transformation of developmental indicators [26]. If a state had only one Aspirational district, a district with higher anemia prevalence as per the National Family Health Survey (NFHS, 2015–16) was selected [1]. The same criteria were considered while choosing two districts of a state having more than two aspirational districts. In addition to the above, the presence of partner agencies (who would conduct the field assessment) was also considered in finalizing the districts by following the study criteria. Recommendations from the union and state governments were also taken into account while finalizing the districts. A total of 58 districts were selected for the assessment (S1 Table in S1 Data). Within each district, two administrative blocks were selected based on the distance of the blocks from the district headquarter. This criterion was adopted with intent to capture supply chain experience of blocks from both semi-urban as well as rural far-to-reach blocks. Accordingly, a total of 116 blocks were covered (S1 Fig).

Further, within each block two health sub-centres (SC) were selected for tracking the distribution of IFA red tablets for pregnant and lactating mothers, two *anganwadi* centres (AWC), courtyard shelters under the Integrated Child Development Services (ICDS), scheme operated by community-level frontline workers (FLW) were selected to track IFA syrup distribution for children 6–59 months and IFA blue tablets for out-of-school adolescent girls (10–19 years). The sample also included two government schools from each block to study the last mile delivery issues with the education department. In particular, the study team visited one primary school to examine supplies for IFA pink tablets for children (5–9 years) and one secondary to study the experience of distribution in IFA blue tablets for adolescent boys and girls (10–19 years). The selections were made in consultation with the district and block-level officials. The blocks were selected based on consultation with district and block officials, but schools, facilities and AWCs were selected randomly. A total of 232 SCs, 232 AWCs, and 116 primary schools and 116 secondary schools were covered for the assessment.

Based on the type of IFA supplement and the target beneficiary group, the interview schedule was designed to capture line department-specific details. Various officials, program staff and health workers were interviewed at the state, district, block, SC, AWC and school levels. The designation of stakeholders identified for the interviews is presented in S2 Table in S1 Data. The survey data was collected through KoBo collect tool. The interviews aimed at understanding the roles and responsibilities of the officials in various components of the supply chain management such as forecasting, procurement, distribution and last mile delivery. The interview also enquired about the data and methods used for demand estimation as well as procedures adopted for procurement, warehousing and distribution of the IFA supplements. Perception of the stakeholders was also obtained on issues such as quality of procured IFA supplements and timeliness of supplies as well as situation stock availability of IFA supplements round the year. In addition, unit costs for the various IFA supplements for each state is estimated based on the procurement budget and quantities approved under the Record of Proceedings (ROPs) for the year 2018–19 for the National Health Mission (NHM) budget for the respective states (https://nhm.gov.in/).

Descriptive statistical analysis is used to examine the patterns in unit costs as well as other important parameters of the assessment. Lead time i.e., the time taken to complete a cycle of IFA procurements and distribution (from forecasting to last mile distribution) is estimated based on the information received on the time requirements and lags at each stage. The IFA supplementation coverage is assessed for the year 2018–19 based on the Health Management Information System (HMIS) indicators (https://hmis.nhp.gov.in/#!/). In particular, the following indicators are used: a) HMIS 9.9 for children (6–59 months) provided 8–10 doses (1ml) of IFA syrup (bi-weekly); b) HMIS 23.1 and 23.3 for school-going and out-of-schoolchildren (5–9 years) covered under Weekly Iron and Folic Acid Supplementation (WIFS) Junior; c) HMIS 22.1.1 for adolescent boys and girls (10–19 years) covered under WIFS, and; d) HMIS 1.2.4 for coverage among pregnant women (PW).

The indent gap is estimated as the difference between the estimated target beneficiaries extracted from AMB dashboard (www.anemiamuktbharat.info) and the actual indent estimate used by the states for the procurements. The AMB target has been taken from the drug required for the particular state as per the AMB dashboard. The "indent" and "receipt" data is collected through the information ascertained during the assessment for the actual indent placed and quantity received during the year by the state for each IFA category. If the data was not received from the state, the same has been taken from the HMIS (total stock received for the year) as uploaded by the state.

## Results

### IFA supplementation coverage, 2018–19

In 2018–19, the IFA supplementation covered 8.3% children (6–59 months), 14.9% children (5–9 years), 28.0% adolescents (10–19 years) and 84.8% pregnant women (Table 1). The coverage varied with major gaps in coverage of children from schools and AWCs in most of the states. For instance, 23 out of 36 states/UTs reported less than 5% coverage of IFA syrup among children under five. Similarly, 22 states/UTs had less than 5% coverage of IFA pink tables provided under the WIFS Junior component for children 5–9 years. The coverage of IFA supplementation among pregnant women was also a concern and varied from 15.6% in Nagaland to almost full coverage in Assam.

**Table 1:** IFA supplementation coverage by beneficiary groups by states/UTs, HMIS 2018–19.

States / UTs	IFA Red, Pregnant Women	IFA Pink, Children (5–9 years)	IFA Blue, Adolescents (10–19 years)	IFA Syrup, Children (6–59 months)	Mean coverage
A & N Islands	72.3	0.0	0.0	11.9	21.1
Andhra Pradesh	95.0*	95.0*	95.0*	11.8	74.2
Arunachal Pradesh	44.8	3.7	4.6	0.0	13.3
Assam	95.0*	52.9	49.9	22.2	55.0
Bihar	69.0	0.0	0.4	0.0	17.4
Chandigarh	90.2	0.0	26.2	2.2	29.7
Chhattisgarh	95.0*	2.5	48.4	5.4	37.8
D&N Haveli	85.9	95.0*	95.0*	68.8	86.2
Daman & Diu	95.0*	24.4	32.0	0.3	37.9
Goa	72.5	0.0	39.9	0.0	28.1
Gujarat	95.0*	27.3	36.7	19.7	44.7
Haryana	59.5	0.0	51.9	3.0	28.6
Himachal Pradesh	79.9	1.1	6.6	10.7	24.6
Jammu and Kashmir	35.5	0.2	0.2	0.7	9.2
Jharkhand	93.1	13.3	31.8	2.0	35.1
Karnataka	95.0*	2.4	11.6	2.3	27.8
Kerala	94.3	0.0	37.3	0.6	33.1
Lakshadweep	95.0*	0.0	0.0	0.0	23.8
Madhya Pradesh	92.1	57.9	48.9	16.7	53.9
Maharashtra	94.2	25.8	57.5	7.9	46.4
Manipur	38.8	4.1	3.5	0.1	11.6
Meghalaya	33.5	0.0	23.5	0.4	14.4
Mizoram	39.4	5.2	42.2	0.2	21.8
Nagaland	15.6	0.5	28.6	0.1	11.2
NCT of Delhi	51.3	0.0	0.0	0.1	12.9
Odisha	82.8	23.3	28.9	17.9	38.2
Puducherry	48.5	4.3	62.8	6.9	30.6
Punjab	55.0	18.7	48.0	9.6	32.8
Rajasthan	45.2	1.7	3.5	4.0	13.6
Sikkim	69.7	1.5	72.8	2.1	36.5
Tamil Nadu	95.0*	21.8	79.9	3.2	50.0
Telangana	95.0*	0.0	1.4	0.0	24.1
Tripura	56.5	0.9	7.9	0.0	16.3
Uttar Pradesh	95.0*	14.8	16.0	0.4	31.6
Uttarakhand	31.8	0.8	4.8	0.1	9.4
West Bengal	88.8	7.5	10.2	55.8	40.6
All India	84.8	14.9	28.0	8.3	34.0

Source: Calculated by authors (based on HMIS Indicators).

*Estimated coverage is pegged at a ceiling of 95%.

### IFA supply chain lead time index

Lead time is a fundamental indicator to gauge the efficiency of a supply chain. This indicator represents the total time taken by the state from forecasting to the actual delivery to the last mile (delivery points like School, Sub Center, and Anganwadi). The shorter the lead time the more efficient the supply chain. The timeline for various components of the supply chain activities is collected from the State Officials in the National IFA Supply Chain Diagnostic Assessment, 2019. The total lead time (in weeks) is the sum of the total lead time of each activity involved–indent compilation, tender/purchase order, procurement, distribution till districts and the last-mile delivery. The lead time index varies from 18 weeks in Goa to 62 weeks in Uttarakhand (Table 2). Most of the states require 6–8 months to complete a supply chain cycle. In terms of the various components of the supply chain, the process of indent compilation and last-mile delivery takes relatively lesser time than other aspects. With a few exceptions (Rajasthan and Chhattisgarh), indent compilation in most states is completed within a month.

**Table 2:** Lead time index (in weeks) for IFA supplements supply chain by states / UTs, 2018–19.

States	Indent Compilation	Tender/Purchase order	Procurement and delivery	Block-level Distribution	Last Mile Delivery	Lead Time
Goa	2	8	5	2	1	18
Sikkim	2	3	7	3	4	19
Telangana	4	4	8	4	2	22
Meghalaya	4	1	12	4	2	23
Haryana	2	4	15	2	1	24
Mizoram	2	12	8	1	1	24
Uttar Pradesh	4	9	8	2	1	24
Maharashtra	4	12	7	2	1	26
Delhi	5	5	10	4	2	26
Nagaland	4	2	6	12	4	26
Kerala	4	12	8	4	1	29
Tamil Nadu	4	12	8	4	1	29
Jharkhand	4	12	10	4	1	31
Manipur	4	12	10	4	1	31
Punjab	4	12	12	2	1	31
West Bengal	1	4	19	1	6	31
Odisha	1	15	10	6	2	34
Rajasthan	8	12	6	4	4	34
Madhya Pradesh	2	12	8	12	2	36
Tripura	4	12	12	8	1	37
Bihar	3	13	10	11	4	41
Karnataka	1	22	11	11	1	46
Andhra Pradesh	4	12	3	28	1	48
Arunachal Pradesh	4	3	12	28	1	48
Gujarat	3	5	16	23	1	48
Chhattisgarh	12	20	15	2	2	51
Assam	2	7	17	22	1	53
Himachal Pradesh	1	23	8	16	8	56
Uttarakhand	4	35	11	11	1	62
Average time in weeks	4	11	10	8	2	35

Source: Calculated by Authors (based on primary data)

Note: The average estimated is rounded off to the nearest decimal.

Similarly, the last mile delivery usually takes about 2–4 weeks though it can be higher in certain hilly regions. The tendering process for procurements (quotations and selection of suppliers) is completed in about three months though certain inter-state variations are noted. In some states (like Chhattisgarh, Himachal Pradesh, Karnataka and Uttarakhand), the process of finalizing a purchase order requires more than four months. Procurement and delivery to designated warehouses also take about 2–3 months. Block-level distribution is completed within a month though it is a major problem in states with hilly terrain or poor road infrastructure.

### Forecasting requirement of IFA supplements

The forecasting practices vary across different states. While 18 out of 29 states consider indents compiled from lower levels, only ten states also consider stock-at-hand for arriving at estimates (Table 3). Overall, 23 out of 29 States relied on population targets for arriving at the annual requirement. The volume of stock distributed in the previous year was only considered in 11 states. Only 13 states used consumption data for arriving at annual requirements. Although the final estimation of the IFA requirement is done at the State level, 39 out of the 58 districts carried out forecasting of their annual requirements. In comparison, the remaining 19 districts did not forecast their IFA requirements. Out of these 39 districts in which forecasting was being done at their level, 32 districts considered population targets for forecasting. Only 20 districts consider stock-at-hand while calculating requirements. Similarly, stocks distributed in the previous year were considered in 20 districts.

**Table 3:** Factors considered while forecasting the procurement requirements for IFA supplements across states / UTs, 2018–19.

State	Stock Distributed in the previous year	Consumption data from lower level	Indents from lower level	State/District targets	Stock at Hand
Andhra Pradesh	Yes	No	No	No	No
Arunachal Pradesh	No	No	Yes	Yes	No
Assam	Yes	Yes	No	Yes	Yes
Bihar	No	No	Yes	Yes	No
Chhattisgarh	No	No	Yes	Yes	No
Delhi	No	Yes	No	Yes	No
Goa	Yes	Yes	Yes	Yes	Yes
Gujarat	Yes	Yes	Yes	No	Yes
Haryana	Yes	Yes	Yes	Yes	Yes
Himachal Pradesh	Yes	Yes	Yes	Yes	Yes
Jharkhand	No	No	No	Yes	No
Karnataka	Yes	Yes	Yes	Yes	Yes
Kerala	No	Yes	Yes	Yes	Yes
Madhya Pradesh	No	No	No	Yes	No
Maharashtra	Yes	Yes	Yes	Yes	Yes
Manipur	No	No	No	Yes	No
Meghalaya	No	No	Yes	Yes	No
Mizoram	No	No	No	Yes	No
Nagaland	No	No	No	Yes	No
Odisha	No	No	No	Yes	No
Punjab	Yes	No	No	Yes	No
Rajasthan	No	Yes	Yes	No	No
Sikkim	No	No	Yes	Yes	No
Tamil Nadu	No	No	Yes	Yes	Yes
Telangana	Yes	Yes	Yes	Yes	Yes
Tripura	No	Yes	No	No	No
Uttar Pradesh	No	No	Yes	No	No
Uttarakhand	No	No	Yes	Yes	No
West Bengal	Yes	Yes	Yes	No	No

Source: based on primary data

Only 16 districts considered consumption data from the lower level as well. At the administrative block level, too, there was a lack of clarity regarding the type of data to be used for forecasting indent. 58% of the blocks used population targets, while 47% also used consumption data from peripheral facilities and 33% considered stock distributed in the previous year. Almost all states forecast the IFA requirements annually. In the case of IFA red tablets, only Haryana and West Bengal opted for half-yearly and quarterly forecasting. In the case of IFA blue, only Bihar did half-yearly forecasting. In 6 states, IFA pink was yet to be rolled out for the year 2018–19. Forecasting for IFA pink was being done annually in 25 states, with Haryana and West Bengal opting for half-yearly forecasting.

### Indent gap for IFA supplements

This indicator attempts to highlight the gap between the drug requirement as per the population targets and the actual procurement of each IFA category. The indent gap is the difference between the AMB target and the indent. The indent gap is usually large in most of the states (S3 Table in S1 Data). On average, the indent gap is 60% for IFA red tablets, 75% for IFA pink, 50% for IFA blue and 70% for IFA syrup. The indent gap is higher for IFA supplementation among under-five or 5–9 years children. This is attributable to the low coverage of these components of the IFA supplementation program. The coverage of IFA red and IFA blue is relatively high. Besides, the indent is usually based on the past experience of IFA distribution. Indent gaps are usually higher in contexts where supplementation coverage is lower.

Also, the indent gap can be 100% in cases where the program is not functional or if the stocks from the previous year are available to be carried over to the next financial year. Out of the 29 states, only five states–Goa, Haryana, Delhi, Rajasthan and Telangana did not procure for IFA Red for 2018–19. This was due to excess stocks lying in the warehouses which needed to be utilized. Apart from these, around 14 states had an indent gap of more than 60% as per the AMB dashboard target. Odisha had zero gap followed by Mizoram, procuring 3.4% more than the AMB target. Whereas Uttarakhand was the only outlier with their indent being three times that of the AMB target.

### Procurement

A total of 18 states (Andhra Pradesh, Bihar, Chhattisgarh, Delhi, Gujarat, Haryana, Jharkhand, Karnataka, Kerala, Madhya Pradesh, Maharashtra, Odisha, Punjab, Rajasthan, Tamil Nadu, Telangana, Uttar Pradesh, West Bengal) have a separate procurement agency to procure its drugs as per the indent received from the line department for health and AMB. States like Arunachal Pradesh, Assam, Goa, Himachal Pradesh, Manipur, Meghalaya, Mizoram, Nagaland, Sikkim, Tripura and Uttarakhand do not have a separate procurement agency. Procurement in these states is done by the procurement division of the State NHM or Directorate of Health Services (DHS). Procurement for IFA is done at the State level in all states except Himachal Pradesh where the district procures IFA locally at their level. Out of the 29 States visited, a total of 23 States reported that they have Standard Operating Procedure (SOPs) / Guidelines that are followed for the procurement of drugs. 26 out of the 29 States visited undertake independent quality testing apart from the quality test report submitted by the supplier.

### Unit cost of IFA supplements

The unit cost for each IFA has been estimated based on the ROP/PIP 2018–19. Table 4 shows the estimated unit costs for various states. The estimates are taken from the ROP/PIP document of the state, in some state the cost of IFA supplies is missing in the document, therefore not reflected here. The unit costs for IFA tablet formulations are more or less similar across states. Unit cost of IFA red varies from Indian National Rupee (INR) 0.12 in Gujarat to INR 1.17 in West Bengal. Unit cost of IFA blue varies from INR 0.14 in Tamil Nadu to INR 0.48 in Mizoram. Unit cost of IFA pink varies from INR 0.10 in Tamil Nadu to INR 1.28 in Assam. The unit cost of IFA syrup varies from INR 5.72 in Telangana to INR 15 in Sikkim and Mizoram. The unit costs are relatively higher for states located in the Himalayan foothills in India, including the northeastern region, and other states with substantial hilly terrains.

**Table 4:** Estimated unit cost of IFA supplements (tablets / syrups) by states / UTs, 2017–18.

State	IFA Red	IFA Blue	IFA Pink	IFA Syrup
Andhra Pradesh	0.18	0.17	0.14	-
Arunachal Pradesh	0.30	0.30	0.13	6.04
Assam	0.18	0.20	1.28	6.04
Bihar	-	0.16	0.13	6.04
Chhattisgarh	0.30	0.30	-	6.04
Delhi	0.32	0.32	0.12	6.37
Goa	-	0.40	0.13	6.04
Gujarat	0.12	0.19	0.10	8.41
Haryana	-	0.17	-	6.07
Himachal Pradesh	0.23	0.23	0.12	12.07
Jharkhand	0.30	0.15	0.28	7.00
Karnataka	0.30	0.28	-	6.04
Kerala	0.35	0.30	0.13	6.03
Madhya Pradesh	1.00	0.15	0.13	6.03
Maharashtra	0.20	0.30	-	-
Manipur	0.26	0.38	0.13	6.04
Meghalaya	0.31	0.26	0.12	6.04
Mizoram	0.40	0.48	1.00	15.00
Nagaland	1.00	0.40	-	6.00
Odisha	0.16	0.16	0.13	6.04
Punjab	0.15	0.15	0.13	6.04
Rajasthan	-	0.17	0.12	6.66
Sikkim	0.40	0.26	1.00	15.00
Tamil Nadu	0.30	0.14	0.10	6.03
Telangana	-	0.16	0.13	5.72
Tripura	0.15	-	-	9.84
Uttar Pradesh	0.15	0.15	0.13	6.03
Uttarakhand	0.25	0.25	0.25	8.00
West Bengal	1.17	0.32	1.10	6.35
Average	0.35	0.25	0.31	7.30

Note: Estimated by authors based on ROP 2017–18.

### Warehousing and inventory management

Among the 29 states, in 19 states (Nagaland, Tripura, Bihar, Manipur, Gujarat, Haryana, Goa, Madhya Pradesh, Telangana, Meghalaya, Uttarakhand, Jharkhand, Karnataka, Chhattisgarh, West Bengal, Sikkim, Punjab) the IFA distribution was not done through State warehouse and instead it was delivered directly to the districts by the supplier (S4 Table in S1 Data). Hence, the state level warehouse did not play any role in distribution of IFA supplies and did not maintain any buffer to meet the interim requirements. In nine out of the 19 states, staff at state warehouses was trained in storage and distribution practices. In six out of the 19 states where IFA is distributed through State warehouse, guidelines for handling drugs were available. Only one out of the 19 states, where IFA is distributed through state warehouse, had the certificate of good warehousing practices and the certificate was provided by the state health department.

Five states (Bihar, Gujarat, Haryana, Sikkim and Tripura) were maintaining a minimum inventory level at the State warehouse. In 14 of the states, no minimum inventory levels were being maintained. In the remaining ten states, IFA was delivered directly at District warehouses by the supplier. Only eight districts out of the 58 districts visited had guidelines for handling drugs available at the district warehouse.

### Transportation and distribution

**Fig 1:**
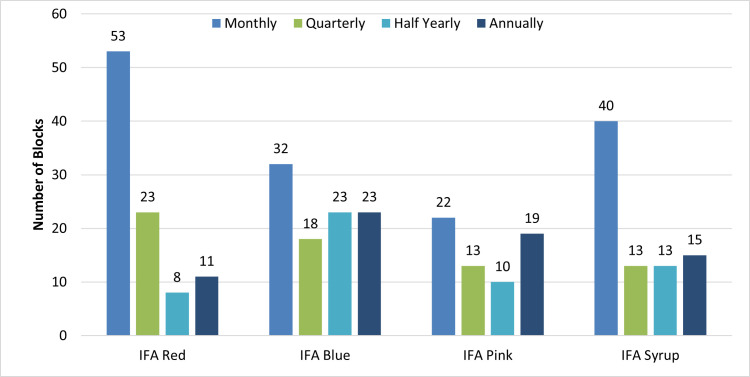
Frequency of IFA distribution from blocks to last-mile delivery facilities, 2019–20.

The frequency of IFA distribution varied across districts (Fig 1). The observations indicate that in 53 blocks out of 116, IFA red tablets were distributed monthly, in 32 blocks IFA blue was distributed annually, IFA pink was distributed annually in 22 Blocks, while IFA syrup was distributed monthly in 40 blocks. Around 54% of AWW/ASHAs responded that they do not request for IFA and are provided supplies by the block with no fixed schedule. 60% of the schools cannot request for IFA and have to wait for the supplies. 28% of the schools requested only when they are out of stock, while 12% cannot request at all. The IFA distribution thus follows a push-based system which is not an ideal approach for the public health supply chain. In addition, in 76% of the Block level facilities, the staff had never received training on storage and distribution. Only 9% reported having received once in the last year. Therefore, it could be concluded that almost there was no storage and distribution training are taking place to manage the proper and timely distribution of IFA supplies.

## Information system

Only 18 states have implemented the Drugs and Vaccine Distribution Management System (DVDMS). DVDMS is a web-based application platform that helps in automated management and analysis of various aspects of the supply chain. It can provide real-time insights on the status of drug availability and other important indicators related to purchases and inventory at various levels. The assessment finds that automated LMIS platforms are available at 20 State Warehouses and 7 Regional Warehouses. At the District level, Logistic Management Information System (LMIS) is present in 19 States, while LMIS was available till Block Level in only ten states. In most states, the district level LMIS is automated, whereas at the block level, it is still to be automated (S5 Table in S1 Data). At the state level, the LMIS updation takes place in Real-time, whereas in most the lower levels the updation is done on a monthly basis (S6 Table in S1 Data).

## Discussion

This assessment identifies the key practices in the management of the IFA supply chain. The IFA supplementation comprises of four drugs namely IFA red tablets (for pregnant and lactating women), IFA blue (for adolescents), IFA pink (for children 5–9 years) and IFA syrup (for children 6–59 months). Each of these product categories have different target beneficiaries and employs different routes for last-mile delivery. It is, therefore, essential to understand the complexity of the combined IFA supply chain and the potential bottlenecks which may hamper the overall efficiency. Each of the major supply chain functions of forecasting, procurement, warehousing and inventory management, transportation and distribution and logistics management information system needed to be analyzed critically to understand the requirements of the supply chain and develop insights and policy recommendations for strengthening of the IFA Supply Chain. The main findings of the assessment are discussed as follows.

### Lack of standardized forecasting process

There was no standard formula for arriving at the annual estimates for IFA drugs. The variations across states show that no uniform forecasting process is being followed. There is a need for a standard forecasting method that should be established across states to ensure a minimum gap between actual demand and procured quantity. The proportion in which each factor was being used to arrive at the final indent was not clearly defined and differed across states. According to AMB guidelines, the state has to estimate 100% consumption and plan for full procurement. Yet, because of wastage and non-consumption, many states do not procure on estimated supplies. Factors like actual program coverage and stock-in-hand were not taken into account for forecasting. The lack of clarity often leads to gaps in estimated demand and actual demand. Lack of availability of consumption data was also a major bottleneck in ascertaining the validity of these estimates. Although districts prepare and submit indents at their level, the final estimates are made on the basis of population figures. The review of the forecasted estimates and supplies estimation by a specific department is missing in most cases. Apart from this, the forecasting is majorly affected by the estimated denominators (target beneficiaries) which is a major concern.

### Complexity in procurement

With the provision for e-tendering or online bidding, the procurement agencies have ensured transparency and have limited interference in the procurement process. The main objective of this setup was to procure drugs at minimum cost and ensure timely delivery. But the procurement process is a lengthy, time-consuming task and prone to procedural delays. It has the maximum contribution in the total lead time for the IFA supply chain. While populated states find suppliers ready to deliver drugs at low costs, a number of smaller states especially the Northeastern states find it difficult to procure at lower costs. This is due to a low drug requirement and partly due to transportation costs involved in delivering across difficult-to-reach terrains. Therefore, these states have to rely on supplies from Central public sector units (PSUs), which are often delayed and results in stock-outs at health facilities. This activity also gets delayed because of absence of certified labs in the vicinity. The procurement for IFA in most of the States is an annual affair with the single lowest bidder being selected for procurement. However, dependency on a single lowest bidder may result in delays if the supplier defaults or cannot meet the commitments due to capacity constraints. There is no backup option in such a case and the whole supply chain is affected adversely. Another issue in the procurement process was the delay in releasing funds which affects the procurement process adversely. It also affects the relationship with the suppliers who are more willing to supply to states where payment is made promptly or within the stipulated time.

### Mismanaged inventories

It is imperative that inventories are managed in most cost-efficient manner. Storage costs, holding and carrying costs, loss due to pilferage have to be minimized while maintaining the service level and expected customer response. In most of the CHC and Block level facilities basic inventory management techniques like maintaining minimum inventory level, reorder level, average monthly consumption and estimation of lead time were not in practice. SOPs/Guidelines for warehouse and inventory management were not available. Besides, staff involved in the storage and management of inventory is found to be lacking in training and capacities. The warehouses were usually understaffed with insufficient staff/helpers for handling and loading stocks at warehouses. Another major issue health facilities face is the lack of proper storage, especially at the Block or CHC level. The limited storage available at CHCs is used for vaccines and other essential drugs. IFA supplies being a supplementation drug are usually stored in inappropriate spaces (such as underneath a staircase). Improper storage is a major risk for damage to drugs thereby rendering it unsuitable for further distribution. All these issues contribute to inefficiencies in service delivery and costs.

### Ad hoc distribution

Transportation and distribution of IFA supplements is a major concern in most States and is affected by two main factors–a) IFA is considered as a program drug not an essential drug and thus receives less priority b) lack of planning to ensure regular distribution. IFA supplies are generally clubbed with other drugs and are adjusted as per the available space in the transport vehicle. Inadequate availability of transport vehicles often resulted in delays. In the absence of a definite schedule or plan of distribution, the supplies are made on an ad hoc basis or on the basis of stock availability. Further, the distribution and timing of supplies to health facilities was not based on the request or requirement from lower levels but instead on the delivery of stocks from the supplier and on the availability of transport vehicles. The distribution system for IFA drugs does not follow a pull-based (demand-driven) mechanism. It was also observed that the quantity distributed is not based on requirement but is instead distributed in equal amount across all facilities. Flexibility in distribution at the block level is desirable to ensure quick responses to cater to demand at the service delivery points but these decisions have to be based on some logical thinking. However, having a fixed distribution is a must for streamlining supplies with a clear idea on the expectations and role of all stakeholders in the supply chain.

### Barriers in information flow

The Logistics Management Information System (LMIS) in public health supply chains in developing countries like India generally suffers from the absence of an integrated Management Information System (MIS). The problem is reflected in the case of the current DVDMS (Drug and Vaccine Distribution System) platform as well. With the exception of two southern states of Tamil Nadu and Kerala, other states had DVDMS platform implemented till the district level and some states have it till health facility level, though not updated on a real-time basis. The mode of MIS is manual and data updation is done on a monthly basis which further limits the availability of real-time data. It is observed that at the peripheral levels, the stock registers are not maintained properly which makes it impossible to retrieve reliable consumption data.

### Inadequacies at the last mile

The practices related to the last mile delivery varied from state to state, especially in case of IFA blue and IFA pink. In few states like Tamil Nadu and Kerala, the village health nurse (VHN) delivered the IFA supplies to schools while in other states, the IFA blue and pink supplies were delivered to the block education department and the nodal teacher then collects the IFA supplies during monthly meetings at the education department. It was observed that the delivery using the health workforce like village health nurse facilitated better monitoring of the program. It also minimized the dependency on education department for reporting. While in states where delivery had to be made through the education department, there were delays in collecting reports. There is a need for better coordination between the two departments to ensure availability of timely reports and stock data. Again, absence of an automated information system makes the system dependent on manual reports. Manual reporting is often delayed, prone to manual errors, and does not provide Real-time data, making monitoring the supply chain difficult. Absence of manual reporting formats/forms at peripheral level led to further delays and inaccurate program coverage data.

### Study limitations

There were some limitations to the IFA supply chain assessment. The assessment covered all the 29 states but excluded union territories (UTs). The assessment covered only two districts in each state which gave an indicative picture of the supply chain practices but a larger number would have further substantiated the findings. The concept of an integrated supply chain encompasses both extremes of the supply chain; however, for this assessment, manufacturers and suppliers were not included as it focused more on supply chain management. The assessment focused on the functional areas of the supply chain like forecasting, procurement, inventory management, and transportation and information system. The lead time and its component are the assessed only for one year (2018–2019), it is not representing the lead time for all the years. The assessment of lead time must be ensured every year to compare either it is improving or not. Finally, budgeting, financing and supply chain costs were not part of the assessment.

### Agenda for correction action

Building flexibility, diffused accountability, reducing multiple levels of the supply chain, and insertion of well-trained human resources are necessary for effective management of the public health supply chain [27]. It is also necessary to understand the interaction of health system elements like governance, financing, and information system with the supply chain and their effect on the coverage, quality, efficiency, and equity of the services. Program managers should be equipped with geo-visual analytics that integrates health and supply chain indicators with geographic mapping to provide a system-wide view [28]. Lack of timely, accurate and reliable information can render any supply chain ineffective [29]. The IFA supply chain suffers in the absence of an efficient information system. Linking long-term planning with a monitoring and evaluation framework and tactical indicators can enable evidence-driven decisions. An ideal health supply chain would integrate people and processes with adequate technology [30].

The efforts on strengthening the public health supply chain in India have primarily focused on the upper echelons of the supply chain. These reforms were done on two fronts. The first was to streamline the procurement process to ensure timely procurement of drugs. The second was to ensure warehousing infrastructure at the state level. States have set up procurement agencies with the standardized process, e-tendering and bidding. State and regional warehouses have also been set up to ensure coverage to all districts. However, supply chain management at the Block or village level has remained untouched. The supply chain at these levels is shared by different drugs with each having specific requirements for its delivery to the beneficiaries. Operational efficiencies at the block or village level are critical to the success of the supply chain. In the case of IFA drugs, the IFA blue has a substantially different delivery requirement than IFA red or even IFA syrup. Also, supply chain functions at these levels majorly involve replenishment, distribution and information management. The distribution of IFA blue and pink in most of the state it is conducted through education department other than only the health department used to supply. The concerned officials of the education department are supposed to distribute across the schools. Hence, there is a difference in the distribution last mile delivery mechanism for IFA blue and pink.

Based on the findings, below are key policy recommendations that may help improve the efficiency of the IFA supply chain. The agenda includes changes that are needed to reform the IFA supply chain as well as strategies to improve operational performance within the current system.

- The upper echelons of the supply chain are connected through the DVDMS system while the block level and below facilities are not connected with this system and instead has to depend on a manual recording system. There is a need to connect the block level facilities and the service delivery points (SDPs) like Anganwadi, Schools and sub-centers with the main DVDMS system to make real-time information sharing possible. This would facilitate timely availability of information, quick response of stock-outs at SDPs, beneficiary coverage, minimum errors and informed decision making at all levels.
- Inter-department coordination (between Health, RBSK and Education department) was also one of the challenges observed for the IFA supply chain. It would be good to have a state and district task force for AMB to represent RBSK, Education, and other line departments.
- It was observed that the store managers and pharmacist in charge of the warehouse were not trained in inventory management concepts. Basic concepts like minimum inventory level, reorder level, average monthly consumption was not employed while making decisions regarding supplies. Staff at each level should be introduced to the system concepts and trained to manage inventory replenishment, distribution, and information flows. Every person involved in the chain must be able to understand their individual role.
- The unavailability of transport vehicles was seen as a standard issue at most of the levels. This results in delays in distribution, higher costs and stock-outs. A Specialized third-party logistics provider can be employed by the state and district program teams to take care of the delivery of drugs. In developing countries, case studies on outsourcing transportation for vaccines have shown positive results in achieving better efficiency at minimized costs [31].
- Continuous monitoring is important through monthly/quarterly review meetings at the district level. Program Officers should conduct a review meeting of each block based on their stock distributed, stock availability, and stock out incidences. Timelines should be fixed for corrective actions and followed up in subsequent meetings to maximize the efficiency of supply chain processes.

## Conclusion

The IFA supply chain assessment has brought forth many issues in the supply chain. It has re-established the need for an action plan to iron out the inefficiencies present in the current setup. The systematic assessment of each functional area gives us a clear understanding of the bottlenecks at each level. It would require active leadership and involvement from all the stakeholders to draw out a road map to strengthen the IFA supply chain. Clear and well-defined interventions focusing on specific bottlenecks need to be developed and pursued with rigor. There is a need for practical and viable solutions that can be implemented in the field. Academic and research institutions and development partner agencies need to collaborate and combine their expertise to come out with a clear set of actions and interventions that would ensure the delivery of essential health and nutrition services to the last beneficiary.

Further research in this area should include the development of a supply chain review framework for measuring the performance on both cost and service level efficiency. A performance matrix may be developed for each level customized to the individual requirements at each level. Measurement of key performance indicators and their determinants would also help streamline the policy efforts.

## Supporting information

S1 Fig Overview of visit plan to administrative facilities for IFA supply chain assessment across states, 2018–19. (TIF)

S1 Data Supplementary data tables are available at PLOS One online. (DOCX)
