# Supplementary figures and images for: Public health supply chain for iron and folic acid supplementation in India: Status, bottlenecks and an agenda for corrective action under Anemia Mukt Bharat strategy
Source: PLoS One. 2023 Feb 24;18(2):e0279827. doi: 10.1371/journal.pone.0279827 (PMC9955604; doi:10.1371/journal.pone.0279827)

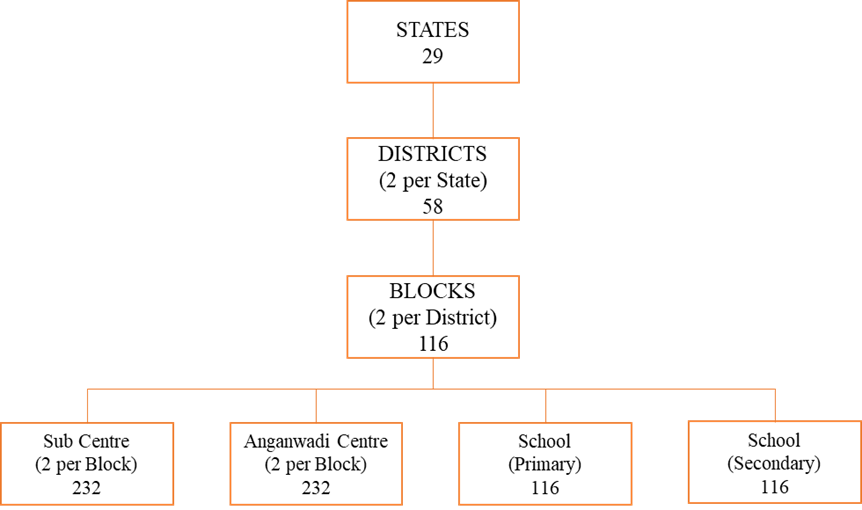

Supplement: S1 Fig — (TIF) [file pone.0279827.s001.tif]
