# Supplementary material for: Public health supply chain for iron and folic acid supplementation in India: Status, bottlenecks and an agenda for corrective action under Anemia Mukt Bharat strategy
Source: PLoS One. 2023 Feb 24;18(2):e0279827. doi: 10.1371/journal.pone.0279827 (PMC9955604; doi:10.1371/journal.pone.0279827)
Supplement: S1 Data — (DOCX) [file pone.0279827.s002.docx]

Supporting Information

**S1 Table. State wise list of districts covered in the supply chain assessment.**

| **State** | **Districts** | |
| --- | --- | --- |
| Andhra Pradesh | YSR (Kadapa) | Vizianagaram |
| Arunachal Pradesh | Namsai | Changlang |
| Assam | Dibrugarh | Baksa |
| Bihar | Vaishali | Purnea |
| Chhattisgarh | Dantewada | Bastar |
| Delhi | Tilak Nagar | Central Delhi |
| Goa | North goa | South goa |
| Gujarat | Kutch | Dahod |
| Haryana | Mewat | Rewari |
| Himachal Pradesh | Shimla | Chamba |
| Jharkhand | West Singhbhum | Dumka |
| Karnataka | Raichur | Yadgir |
| Kerala | Kozhikode | Wayanad |
| Madhya Pradesh | Barwani | Vidisha |
| Maharashtra | Washim | Nandurbar |
| Manipur | Chandel | Imphal west |
| Meghalaya | Ri-Bhoi | East Garo Hills |
| Mizoram | Kolasib | Mamit |
| Nagaland | Kiphire | Wokha |
| Odisha | Nabarangapur | Bolangir |
| Punjab | Firozpur | Moga |
| Rajasthan | Banswara | Baran |
| Sikkim | North Sikkim | West Sikkim |
| Tamil Nadu | Ramanathpuram | Virudhunagar |
| Telangana | Adilabad | Khammam |
| Tripura | West Tripura | Dhalai |
| Uttar Pradesh | Meerut | Chandauli |
| Uttarakhand | Haridwar | Udham Singh Nagar |
| West Bengal | Dakshin Dinajpur | Malda |

**S2 Table. List of officials and stakeholders interviewed across States / UTs, 2018-19.**

| **Component** | **State** | **District** | **Block** | **Sub-center** | **ASHA/**  **AWW** | **School** |
| --- | --- | --- | --- | --- | --- | --- |
| Organization Structure | MD, NHM | Civil Surgeon  District RCHO  District Health Officer | Medical Officer | ANM | ASHA | Nodal  Teacher |
|  | Deputy Director Health Services |  |  |  | AWW | Principal |
|  |  |  |  |  |  |  |
| Forecasting | Nodal Officer, NHM | District Program Officer | Block Program Manager |  |  |  |
|  | State Program Officer | District Data Manager | Block RCHO |  |  |  |
|  | RCH Officer |  |  |  |  |  |
|  | RKSK Officer |  |  |  |  |  |
| Procurement | State Procurement Officer | Medical Officer-In-Charge |  |  |  |  |
|  | Procurement Manager |  |  |  |  |  |
|  | Director, State Medical Service Corporation |  |  |  |  |  |
| Warehousing & Inventory Management | State Warehouse Manager | District Pharmacist | Pharmacist |  |  |  |
|  | Regional Warehouse Manager | Warehouse Manager | Warehouse Manager |  |  |  |
|  | Pharmacist, State Warehouse |  |  |  |  |  |
| Transportation & Distribution | State Warehouse Manager | District Pharmacist | Pharmacist |  |  |  |
|  | Regional Warehouse Manager | Warehouse Manager |  |  |  |  |
|  | Pharmacist, State Warehouse |  |  |  |  |  |
| Logistics Management Information System | State Data Manager | District Data Manager | Block Data Entry |  |  |  |
|  | HMIS Consultant | District Accounts Manager |  |  |  |  |
|  | Procurement Consultant |  |  |  |  |  |
|  | Warehouse Manager |  |  |  |  |  |

**S3 Table. Indent gap (in %) for various IFA supplements procurement done by states / UTs, 2018-19.**

| **States** | **Red** | **Pink** | **Blue** | **Syrup** |
| --- | --- | --- | --- | --- |
| Andhra Pradesh | 93 | 96 | 98 | 39 |
| Arunachal Pradesh | 73 | 96 | 39 | 90 |
| Assam | 71 | 55 | 54 | 81 |
| Bihar | 69 | 68 | 49 | 87 |
| Chhattisgarh | 67 | 100 | -99 | 0 |
| Delhi | 100 | 100 | 98 | 100 |
| Goa | 100 | 100 | 73 | 100 |
| Gujarat | 35 | 46 | 41 | 81 |
| Haryana | 100 | 100 | 26 | 97 |
| Himachal Pradesh | 86 | 73 | 100 | 99 |
| Jharkhand | 39 | 24 | 31 | 83 |
| Karnataka | 100 | 100 | 100 | 75 |
| Kerala | 67 | 100 | 76 | 87 |
| Madhya Pradesh | 55 | 39 | 25 | 64 |
| Maharashtra | 31 | 12 | 49 | 15 |
| Manipur | 30 | 100 | 73 | 51 |
| Meghalaya | 69 | 75 | 32 | 89 |
| Mizoram | -3 | 100 | -32 | 100 |
| Nagaland | 86 | 100 | 12 | 72 |
| Odisha | 0 | 42 | 0 | 0 |
| Punjab | 99 | 97 | 95 | 96 |
| Rajasthan | 100 | 30 | 50 | 64 |
| Sikkim | 76 | 0 | 23 | 50 |
| Tamil Nadu | 43 | 100 | 0 | 62 |
| Telangana | 100 | 100 | 60 | 100 |
| Tripura | 59 | 100 | 100 | 11 |
| Uttar Pradesh | 96 | 97 | 99 | 99 |
| Uttarakhand | -197 | 21 | 80 | 36 |
| West Bengal | 98 | 98 | 93 | 94 |

Source: Calculated by Authors (based on primary data and HMIS data)

**S4 Table. Warehousing features and practices across states / UTs, 2018-19.**

| State | IFA distribution through State/Regional Warehouse | Availability of SOP/Guidelines at the State Warehouse | Certificate of Good Warehousing Practices at State Warehouse | State Warehouse Staff trained on storage and distribution | Minimum stock level is maintained at the State Warehouse |
| --- | --- | --- | --- | --- | --- |
| Nagaland | Yes | No | No | Yes | No |
| Himachal Pradesh | No | NA | NA | NA | NA |
| Tripura | Yes | Yes | No | No | Yes |
| Uttar Pradesh | No | NA | NA | NA | NA |
| Assam | No | NA | NA | NA | NA |
| Bihar | Yes | No | No | Yes | Yes |
| Manipur | Yes | Yes | Yes | Yes | No |
| Gujarat | Yes | Yes | No | No | Yes |
| Haryana | Yes | No | No | Yes | Yes |
| Goa | Yes | No | No | No | No |
| Maharashtra | No | NA | NA | NA | NA |
| Madhya Pradesh | Yes | No | No | No | No |
| Telangana | Yes | Yes | No | Yes | No |
| Meghalaya | Yes | No | No | No | No |
| Uttarakhand | Yes | No | No | No | No |
| Jharkhand | Yes | No | No | No | No |
| Karnataka | Yes | No | No | Yes | No |
| Rajasthan | No | NA | NA | NA | NA |
| Odisha | No | NA | NA | NA | NA |
| Chhattisgarh | Yes | Yes | No | Yes | No |
| Andhra Pradesh | No | NA | NA | NA | NA |
| Tamil Nadu | No | NA | NA | NA | NA |
| West Bengal | Yes | No | No | Yes | No |
| Sikkim | Yes | No | No | No | Yes |
| Mizoram | No | No | No | Yes | No |
| Arunachal Pradesh | No | Yes | No | No | No |
| Delhi | No | NA | NA | NA | NA |
| Punjab | Yes | No | No | No | No |
| Kerala | No | NA | NA | NA | NA |

*NA – Not Applicable

**S5 Table. Availability of automated v/s manual LMIS across States / UTs, 2018-19.**

| State | Type of LMIS at Warehouse | | | |
| --- | --- | --- | --- | --- |
|  | State Warehouse | Regional Warehouse | District Warehouse | Block Warehouse |
| Andhra Pradesh | Manual | NA | Manual | Manual |
| Arunachal Pradesh | Automated | NA | Manual | Manual |
| Assam | Automated | NA | Automated | Manual |
| Bihar | Automated | Automated | Automated | Manual |
| Chhattisgarh | Automated | NA | Automated | Automated |
| Delhi | Manual | NA | Manual | Manual |
| Goa | Automated | NA | Automated | Manual |
| Gujarat | Automated | Automated | Automated | Manual |
| Haryana | Automated | Automated | Automated | Automated |
| Himachal Pradesh | NA | NA | Manual | Manual |
| Jharkhand | Manual | Manual | Manual | Manual |
| Karnataka | Automated | NA | Automated | Automated |
| Kerala | Automated | NA | Automated | Automated |
| Madhya Pradesh | Automated | NA | Automated | Automated |
| Maharashtra | Automated | Automated | Automated | Automated |
| Manipur | Automated | NA | Automated | Manual |
| Meghalaya | Manual | NA | Manual | Manual |
| Mizoram | Manual | NA | Manual | Manual |
| Nagaland | Manual | NA | Manual | Manual |
| Odisha | Automated | NA | Automated | Automated |
| Punjab | Automated | Automated | Automated | Manual |
| Rajasthan | Automated | NA | Automated | Automated |
| Sikkim | Manual | NA | Manual | Manual |
| Tamil Nadu | Automated | NA | Automated | Automated |
| Telangana | Automated | Automated | Automated | Automated |
| Tripura | Automated | NA | Automated | Manual |
| Uttar Pradesh | Automated | NA | Automated | Manual |
| Uttarakhand | Manual | Manual | Manual | Manual |
| West Bengal | Automated | Automated | Automated | Manual |

Source: based on primary data

**S6 Table. Frequency of Stock MIS updation at various levels by States / UTs, 2018-19.**

| State | State Level | Regional Level | District Level | Block Level | Sub centre Level | School Level | Anganwadi Level |
| --- | --- | --- | --- | --- | --- | --- | --- |
| Andhra Pradesh | Monthly | NA | Monthly | Monthly | Monthly | Monthly | Monthly |
| Arunachal Pradesh | Monthly | NA | Monthly | Monthly | Monthly | Monthly | Monthly |
| Assam | Real-time | NA | Real-time | Weekly | Monthly | Monthly | Monthly |
| Bihar | Real-time | Real-time | Monthly | Monthly | Monthly | Monthly | Monthly |
| Chhattisgarh | Real-time | NA | Weekly | Weekly | Monthly | Monthly | Monthly |
| Delhi | Monthly | NA | Monthly | Monthly | Monthly | Monthly | Monthly |
| Goa | Real-time | NA | Real-time | Monthly | Monthly | Monthly | Monthly |
| Gujarat | Daily | Daily | Monthly | Monthly | Monthly | Monthly | Monthly |
| Haryana | Real-time | Real-time | Real-time | Real-time | Monthly | Monthly | Monthly |
| Himachal Pradesh | NA | NA | Monthly | Monthly | Monthly | Monthly | Monthly |
| Jharkhand | Daily | Daily | Weekly | Weekly | Monthly | Monthly | Monthly |
| Karnataka | Daily | NA | Daily | Weekly | Monthly | Monthly | Monthly |
| Kerala | Real-time | NA | Real-time | Real-time | Monthly | Monthly | Monthly |
| Madhya Pradesh | Real-time | NA | Real-time | Real-time | Monthly | Monthly | Monthly |
| Maharashtra | Real-time | Real-time | Real-time | Real-time | Monthly | Monthly | Monthly |
| Manipur | Real-time | NA | Weekly | Monthly | Monthly | Monthly | Monthly |
| Meghalaya | Daily | NA | Daily | Daily | Monthly | Monthly | Monthly |
| Mizoram | Monthly | NA | Monthly | Monthly | Monthly | Monthly | Monthly |
| Nagaland | Monthly | NA | Monthly | Monthly | Monthly | Monthly | Monthly |
| Odisha | Daily | NA | Daily | Daily | Monthly | Monthly | Weekly |
| Punjab | Real-time | Real-time | Real-time | Monthly | Monthly | Monthly | Monthly |
| Rajasthan | Real-time | NA | Real-time | Monthly | Monthly | Monthly | Monthly |
| Sikkim | Monthly | NA | Monthly | Monthly | Monthly | Monthly | Monthly |
| Tamil Nadu | Real-time | NA | Real-time | Real-time | Weekly | Weekly | Weekly |
| Telangana | Real-time | Real-time | Real-time | Daily | Weekly | Weekly | Weekly |
| Tripura | Real-time | NA | Real-time | Monthly | Monthly | Monthly | Monthly |
| Uttar Pradesh | Real-time | NA | Real-time | Weekly | Monthly | Monthly | Monthly |
| Uttarakhand | Daily | Daily | Daily | Daily | Monthly | Monthly | Monthly |
| West Bengal | Real-time | NA | Real-time | Real-time | Monthly | Monthly | Monthly |

NA: Not Applicable
